# The Phytochemical Profile and Anticancer Activity of *Anthemis tinctoria* and *Angelica sylvestris* Used in Estonian Ethnomedicine

**DOI:** 10.3390/plants11070994

**Published:** 2022-04-05

**Authors:** Ain Raal, Marel Jaama, Meeme Utt, Tõnu Püssa, Vaidotas Žvikas, Valdas Jakštas, Oleh Koshovyi, Khan Viet Nguyen, Hoai Thi Nguyen

**Affiliations:** 1 Institute of Pharmacy, Faculty of Medicine, University of Tartu, 50411 Tartu, Estonia; mareljaama@gmail.com (M.J.); meeme.utt@ut.ee (M.U.); nvietkhan@gmail.com (K.V.N.); 2 Department of Veterinary Biomedicine and Food Hygiene, Estonian University of Life Sciences, 51014 Tartu, Estonia; tonu.pyssa@emu.ee; 3 Institute of Pharmaceutical Technologies, Lithuanian University of Health Sciences, 44307 Kaunas, Lithuania; vaidotas.zvikas@lsmuni.lt (V.Ž.); valdas.jakstas@lsmuni.lt (V.J.); 4 Pharmacognosy Department, The National University of Pharmacy, 61002 Kharkiv, Ukraine; oleh.koshovyi@gmail.com; 5 Faculty of Pharmacy, Hue University of Medicine and Pharmacy, Hue University, Hue City 52000, Vietnam; hoai77@gmail.com

**Keywords:** *Anthemis tinctoria*, *Angelica sylvestris*, ethnomedicine, essential oil, polyphenols, anticancer activity

## Abstract

The aerial parts of *Anthemis tinctoria* L. and *Angelica sylvestris* L. and the roots of *A. sylvestris* have been used as traditional anticancer remedies in Estonian ethnomedicine. The aim of this study was to investigate content of essential oils (by gas chromatography) and polyphenolic compounds (using two different methods of high performance liquid chromatography–mass spectrometry (HPLC–MS)) of both plant species, as well as the in vitro anti-cancer effects of their essential oils and methanolic extracts. The average (*n* = 5 samples) yield of essential oils was 0.15%, 0.13%, and 0.17%, respectively. The principal compounds of the essential oil from the aerial parts of *A. tinctoria* were palmitic acid (15.3%), *p*-cymene (12.6%), and *α*-muurolene (12.5%), and *α*-pinene (45.4%), *p*-cymene (15.5%), and *β*-myrcene (13.3%) in aerial parts of *A. sylvestris*, while isocaryophyllene oxide (31.9%), *α*-bisabolol (17.5%), and *α*-pinene (12.4%) were the main constituents in the roots. The most abundant phenolic compounds in aerial parts were the derivatives of caffeic acid, quinic acid, and quercetin; the main compounds in roots of *A. sylvestris* were chlorogenic acid, quinic acid, and naringenin. The strongest anticancer effects were observed in essential oils of *A. sylvestris* roots and aerial parts on human carcinoma in the mouth cells (KB, IC_50_ 19.73 μg/mL and 19.84 μg/mL, respectively). The essential oil of *A. tinctoria* showed a strong effect on KB and LNCaP cells (27.75–29.96 μg/mL). The methanolic extracts of both plants had no effect on the cancer cells studied.

## 1. Introduction

According to the estimation of the World Health Organization, the approximately 12.7 million new cancer cases that occurred globally in 2008 will increase to 21.3 million by 2030 [1]. When plants with expressed anticancer activity used in Estonian ethnomedicine were studied [2], 44 species with potential anticancer properties were elicited, five of which *Anthemis tinctoria* L., *Angelica sylvestris* L., *Pinus sylvestris* L., *Sorbus aucuparia* L. and *Prunus padus* L. had not been previously described with respect to their tumoricidal activities in the scientific literature. Later, the anticancer activity of essential oil and methanolic extract from needles of *P. sylvestris* and bark of *S. aucuparia* were studied [3,4].

The focus of this study was on *A. tinctoria* (golden marguerite) and *A. sylvestris* (wild angelica). Both species, which grow naturally in Estonia, have been used only in local and neighboring folk medicine. Only a few studies have been published on their chemical composition, mainly on the composition of essential oils and polyphenols.

The content of essential oil in the *A. tinctoria* herb is about 0.1% [5]. The main compounds found in the essential oil are *α*-eudesmol, *γ*-cadinol, *γ*-cadinene, decanoic acid, T-muurolol, 1,8-cineole, and *β*-pinene [6,7]. Chlorogenic acid, gentisic acid, and rosmarinic acid, as well as the flavonoids apigenin-7-glucoside, patuletin, and quercetin, have been found in the aerial parts of *A. tinctoria* [8,9]. A new flavonoid, tinctosid, has been identified [10]. Fifteen flavonoid aglycones, twelve glycosides, and one caffeoyl-*O*-flavonoid were identified in the extracts of *A. tinctoria* [11].

The roots of *A. sylvestris* contain 0.16% of essential oil, flowers 0.52%, leaves 0.08%, and seeds of *A. sylvestris* contain 1.1% of essential oil with the main components being limonene, *α*-pinene, myrcene, *β*-fellandrene, *α*-hamrigren, and *β*-sesquifellandrene [12,13,14]. The essential oil of roots contains mostly sesquiterpenoids, while the essential oil of seeds contains monoterpenoids [13,15]. Moderate amounts of phenolic compounds, and a small amount of flavonoids, have been found in the herb of *A. sylvestris* [16], but their exact composition is unknown.

The aim of this study was to investigate the chemical composition of *A. tinctoria* and *A. sylvestris*, as well as the anti-cancer effects of essential oils and methanolic extracts.

## 2. Results

### 2.1. Chemical Composition

#### 2.1.1. Content and Chemical Composition of Essential Oils

The average (*n* = 5 samples) yield of essential oils in the aerial parts of *A. tinctoria* and *A. sylvestris*, and in the roots of *A. sylvestris* was 0.15%, 0.13%, and 0.17%, respectively.The main components of the essential oil from the aerial parts of *A. tinctoria* were palmitic acid (15.3%), *p*-cymene (12.6%), and *α*-muurolene (12.5%), while the content of other compounds was less than 10% (Table 1). Ocimenes (Z and E), isoborneol, crysanthenyl acetate, humulene epoxide, 2-pentadecanone, and nerolidon acetate were found in the essential oil of *A. tinctoria* for the first time. The principal compounds in the essential oil from aerial parts were α-pinene (45.4%), *p*-cymene (15.5%), and *β*-myrcene (13.3%), while isocaryophyllene oxide (31.9%), *α*-bisabolol (17.5%), and *α*-pinene (12.4%) were the main constituents in root oil of the same plant. The essential oil of aerial parts of *A. sylvestris* contained more monoterpenes and cyclic monoterpenes but less sesquiterpenes and bicyclic sesquiterpenes than the oil hydrodistilled from the roots of *A. sylvestris* (Table 2).

#### 2.1.2. Identification of Polyphenolic Compounds

By two methods, 41 phenolic compounds were identified in *A. tinctoria* herb, 21–23 in *A. sylvestris*, and 10 compounds in roots of *A. sylvestris*. The most abundant were derivatives of caffeic acid, quinic acid, and quercetin (Table 3 and Table 4). In contrast, 10 polyphenolic compounds were detected in *A. sylvestris* root extract, the most abundant of which were chlorogenic acid, quinic acid, and naringenin. Substances common for analyzed herbs and roots were naringenin, chlorogenic acid, quercetin, and coumarylquinic acids.

Roots of *A. sylvestris* contain a number of phenolic compounds in remarkable quantities that remain unidentified. Some of these have a UV absorption maximum near 306 nm that refers to hydroxystilbenes such as resveratrol or piceatannol and six compounds have a negative collision fragment with *m*/*z* = 201 (Figures S1–S3).

The principal polyphenols in the aerial parts of *A. tinctoria* were caffeoylquinic (chlorogenic) and dicaffeoylquinic acids as well as several glycosides of quercetin and patuletin, in the aerial parts of *A. sylvestris* there were also chlorogenic and caffeoylquinic acids and different glycosides of quercetin. The principal polyphenols of *A. sylvestris* roots were neo-chlorogenic acid, caffeic acid glucosides, feruloylquinic acid, and naringenin together with naringenin chalcone. However, the total polyphenol content (TPC) of aerial parts of *A.tinctoria* was about 3.1 and 4.5 times higher than the TPC of aerial parts of *A. tinctoria* and roots of *A. sylvestris*, respectively, estimated by comparison of UV chromatogram areas at 280 nm. Total content of flavonol glycosides (TCF) of aerial parts of *A. tinctoria* was about 4 and 8 times higher than TCF of aerial parts of *A. tinctoria* and roots of *A. sylvestris*, respectively, estimated by comparison of UV chromatogram areas at 360 nm, the maximum of UV spectra of flavonols. Consequently, the TPC and, particularly, the TCF of the methanol extract of both aerial parts and especially of roots of *A. sylvestris* was too low to expect remarkable effects on cancer cells.

Phenolic compounds were identified using the selected MRM transition detection method and inhouse database. It was found that aerial parts accumulated most of phenolic compounds while in root samples some compounds were not detected and others were found in lower abundance. The most abundant compounds were phenolic acids (chlorogenic and dicaffeoylquinic acids). Flavonoid aglycones and their glycosides were detected in lower abundance. Qualitative profiles of selected phenolic compounds were similar between samples, although naringenin was detected only in *A. sylvestris* samples, and luteolin was detected only in *A. tinctoria* samples. It should be emphasized that soem of the phenolic compounds, such as neochlorogenic, and chlorogenic acids, rutin, and dicaffeoylquinic acids were confirmed by both LC–MS methods. The presence of caffeic acid, ferulic acid, quercetin, isorhamnetin (detected in the aerial parts of tested species), luteolin, naringenin (found in samples of *A. sylvestris*), and diosmetine derivatives was also confirmed by both methods. The inequality of methods used and the selected methodological conditions was the basis for supplementing the data obtained. The UPLC–MS/MS method provided more detailed data on luteolin derivatives. Luteolin-7-rutinoside was detected in all samples tested by UPLC–MS/MS, while other luteolin derivatives (luteolin-7-glucoside, luteolin-4-glucoside, and luteolin aglycone) were detected only in *A. tinctoria* aerial parts. The UPLC-based triple quadrupole MRM transitions were more abundant for detection of aglycone monomers as free ferulic acid, caffeic acid, and chlorogenic acid (which was detected in all samples), as well as quercetin and isorhamnetin aglycones (which were detected in aerial parts of *A. sylvestris*), and apigenin aglycone (found in all samples). The quercetin derivative quercitrin (quercetin 3-rhamnoside) was detected in all samples by UPLC–MS/MS as against flavone isorhamnetin-3-rutinoside, which was characterized only in the aerial parts of *A. sylvestris*.

Altogether, ion trap enabled us to identify and semi-quantify 47, and triple quadrupole 22, substances at least in one of the studied plant extracts. This difference between results can be explained by the greater versatility of ion trap, but triple quadrupole is in turn more sensitive, allowing lower concentrations of substances to be determined. It is therefore advisable to analyze any plant extract using two different types of mass spectrometry.

#### 2.1.3. Total Content of Different Compounds

Quantitative analysis of polyphenols was performed mainly, with the exception of chlorogenic acid, by substance groups. As can be seen from Table 5 and Table 6, most of the phenolic compounds in the three studied plant materials were quinic acid derivatives (chlorogenic acids, di- and caffeoylquinic acids, and feruloylquinic acid). The second biggest group, which is completely absent in the roots of *A. sylvestris*, were glycosides of different flavonols (quercetin, myricetin, isorhamnetin, kaempferol, and patuletin). Phenolic acid glycosides (caffeic and coumaric acid), flavanons (naringenin and eriodictyol), flavones (luteolin), and stilbenols(pinosylvin) were also represented, mostly in small quantities. In particular, the herb of *A. tinctoria* was distinguished by its abundance in polyphenols both qualitatively and quantitatively. The aerial parts of *A. tinctoria* contain much more total phenolics, total chlorogenic acids, and total flavonols than the herb of *A. sylvestris* whose roots showed the lowest concentrations of the mentioned compounds (Table 5). A similar result was estimated when we analyzed total quinic acid derivatives; the roots studied did not contain flavonols (Table 6). The total phenolics and chlorogenic acids were calculated where the ratio between plant material and solvent was 1:10.

#### 2.1.4. Anticancer Activity of Essential Oils and Methanolic Extracts

The strongest anticancer effects were observed with *A. sylvestris* roots’ and aerial parts’ essential oils on KB cells (IC_50_ 19.73 μg/mL and 19.84 μg/mL, respectively) (Table 7). The same samples showed strong to moderate effects on other cell lines with IC_50_ range of 24.69–38.06 μg/mL. The essential oil of *A. tinctoria* showed a strong effect on KB and LNCaP cells (IC_50_ between 27.75 μg/mL and 29.96 μg/mL, respectively), and a moderate effect on the other cells (IC_50_ ranged from 43.04 μg/mL to 55.45 μg/mL). The methanolic extract of both plants had no effect on cancer cells (IC_50_ > 100 μg/mL). The methanolic extract of *A. sylvestris* roots had a moderate effect on all cancer cells (IC_50_ range: 40.08–66.06 μg/mL).

## 3. Discussion

In previous studies, the essential oil content of *A. tinctoria* inflorescences was 0.10% [5] and 0.10–0.14% [17]. The amount of essential oil obtained in the present study from the aerial parts was slightly higher (0.15%). The content of essential oil in the *A. sylvestris* leaves was 0.08%, in flowers 0.52%, in fruits 1.1%, and in roots 0.16% [15]. In the present study, the content of essential oil found in the herb was about 2 times higher than in the leaves and about 3.5 times lower than in the flowers and similar to the yield of essential oil measured in roots of *A. sylvestris*.

The three main compounds of the essential oil from *A. tinctoria* (palmitic acid, *p*-cymene, and *α*-muurolol (Table 1) were also found in our earlier study of the same species of Estonian origin [7]. They were not identified in the oil from flowerheads of *A. tinctoria* cultivated in the Slovak Republic [6] and were not mentioned among of principal compounds, which were 1,8-cineole (7.9%), *β*-pinene (7.3%), and decanoic acid (5.4%) [18]. Thus, *A. tinctoria* essential oil does not contain just one dominant component, rather, three major components have typically been found in all of these studies. *A. tinctoria* plants seems to be present in several essential-oil chemotypes.

The main compounds in the essential oil of aerial parts and seeds of *A. sylvestris* collected from Serbia were limonene (66.6 and 75.3%) and *α*-pinene (19.0 and 9.6%) [12,14]. In the present study, we did not found limonene, but the concentration of α-pinene (12.4%) was similar to that in the mentioned papers. The content of limonene was lower (5.6%) and the concentration of *α*-pinene higher (25.6%) in the fruits of *A. sylvestris* grown in Turkey. A study by Ağalar et al. (2020) [15] found that the content of limonene in leaves, flowers, fruits, and roots was 0.4–4.2%. The different results from several authors may depend on different varieties (var. *vulgaris* or var. *elatior*) [13]. Thus, the absence of limonene may be influenced by the specific chemotype of *A. sylvestris* growing in Estonia.

In the aerial parts of *A. Tinctoria,* 15 flavonoid aglycones were identified [11]. Similarly to the present study, caffeic acid, rutin, naringenin, chlorogenic acid, patuletin, and quercetin have been previously found in *A. tinctoria* inflorescences. However, apigenin-7-glucoside and rosmarinic acid were not detected in the current study although they were detected by previous authors [8,9].

Many studies report the multiple anticancer properties of plant-derived polyphenols, including inhibitory effects on the proliferation of cancer cells, tumor expansion, angiogenesis, inflammation, and metastasis. Overall, polyphenolic compounds are attractive molecules for cancer treatment [19]. Flavonols quercetin and kaempferol, that are represented mostly by various glycosides derivatives in the aerial parts of both *A. tinctoria* and *A. sylvestris,* have been shown to have various anticancer effects [19]. Quercetin has been reported to reduce both the risk and progression of cancer through free-radical scavenging activity, protecting cells from oxidative stress, inflammation, and DNA damage due to its antioxidant properties and modulating growth of many cancer cell lines by blocking cell-cycle progression and tumor-cell proliferation and by inducing apoptosis [20]. A group of polyphenols that includes apigenin, quercetin, curcumin, resveratrol, EGCG, and kaempferol has been shown to regulate signaling pathways that are central for cancer development, progression, and metastasis [21]. Chlorogenic acid has also shown anticancer activity [22]. The fact that these compounds did not demonstrate an anticancer effect in our tests can be explained by the fact that the extracts tested contained polyphenols, not chlorogenic acids, in various glycosidic forms, which usually have lower biological activities than the corresponding aglycones [23]. It will be necessary to repeat the experiments using enzymatically hydrolyzed plant extracts. Other explanations, for example differences in cancer cell lines, should also be considered.

The strongest anticancer effects, with IC_50_ less than 20 μg/mL, had the essential oils from aerial parts and roots of *A. sylvestris* on KB (human carcinoma in the mouth) cells. All other results showed moderate or weak activity to different cancer cells (Table 5). The same study of ethnomedicinal traditions in cancer therapy [2] also showed the use of chaga mushroom (*Inonotus obliquus*), chamomile (*Chamomilla recutita*), marigold (*Calendula officinalis*), and Scots pine (*Pinus sylvestris*), which have been previously studied in the context of possible anti-cancer effects in vitro. The methanolic chaga muchroom extract exhibited the strongest cytotoxic effects against promyelocytic leukemia cells (HL-60) and lung adenocarcinoma (LU-1, 32.2 and 38.0 μg/mL, respectively), and modest cytotoxic effects against colon adenocarcinoma (SW480), liver hepatocellular carcinoma (HepG2), oral epidermoid carcinoma (KB), and prostate cancer (LNCaP, 41.3–57.7 μg/mL) [24]. The cytotoxic activity of methanolic extract of chamomile (*Chamomilla recutita*) flowers on SK-MEL-2 (melanoma cells, IC_50_ value 40.7 μg/mL) was approximately twofold higher than on KB cells (IC_50_ value 71.4 μg/mL). With the marigold (*Calendula officinalis*) flower extracts, the anticancer activity was more than 100 μg/mL in both cell lines studied [2]. The essential oil from the needles of *P. sylvestris* showed the stronger cytotoxic effect to both negative and positive breast cancer cell lines (MCF7 and MDA-MB231, both IC_50_ 29 μg/mL) than pine methanolic extract (IC50 42 and 80 μg/mL, respectively) [4]. In this context, the potency of the anti-cancer activity of *A. sylvestris* against different cancer cell lines can be considered remarkable.

*α*-Pinene has a weak anti-cancer effect, but its use is limited due to its toxicity to normal body cells [25]. A study of liver carcinoma cells has shown moderate anti-cancer activity of pinene [26]. β-Myrcene has been shown to inhibit specific types of breast cancer cells [27]. In vitro experiments with *α*-bisabolol on pancreatic cancer cells showed strong anti-cancer activity [28]. The rather high concentration of these three terpenoids in the essential oil of *A. sylvestris* is interesting and could have an effect on other cancer cells not yet studied.

To the best of the author’s knowledge, there are no previous studies on the polyphenolic composition of *A. sylvestris*, which has been studied, by us, for the first time. The novelty of the study is also the detection of the anticancer activity of *A. tinctoria* and *A. sylvestris*. Ocimenes (Z and E), isoborneol, crysanthenyl acetate, humulene epoxide, 2-pentadecanone, and nerolidon acetate were found in the essential oil of *A. tinctoria* for the first time. Additionally, the MS analysis was performed by two distinctive methods.

## 4. Materials and Methods

### 4.1. Plant Material

**Figure 1:**
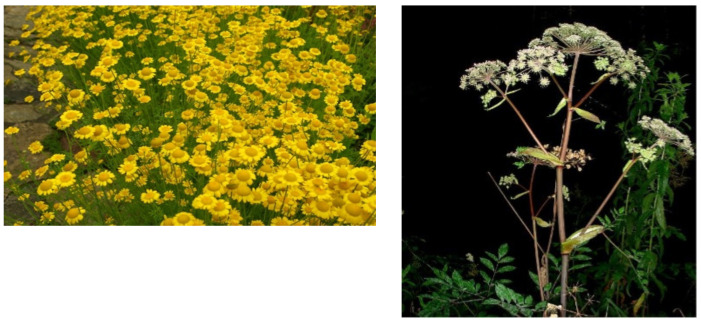
*Anthemis tinctoria* (**left**) and *Angelica sylvestris* (**right**).

The herbs from *A. tinctoria* were collected in July 2019 from Võrumaa, Estonia (57.41594°; 26.504845°). The herbs and roots from *A. sylvestris* were collected in July and September 2019 from Valgamaa, Estonia (57.992805°; 26.110812°) (Figure 1). The plant material was dried in a well-ventilated area at room temperature within 10 days. The stems were separated from the dried plant material, and the remaining mixture of flowers, leaves, and thinner stems was analyzed (195–200 g of each). Voucher specimens have been deposited in Herbarium collection of the Institute of Pharmacy, Faculty of Medicine, University of Tartu under the acquisition numbers AstA.t.1 and ApiA.s.1.

### 4.2. Hydrodistillation of Essential Oil

The essential oils were isolated from dried aerial parts of *A. tinctoria*, as well as from aerial parts and roots of *A. sylvestris* by the modified hydrodistillation method described in the European Pharmacopoeia [29] using 20–45 g of plant material, a 500 mL round-bottomed flask, and 400 mL water as the distillation liquid, and 0.5 mL of xylene in the graduated tube was added to take up the essential oil. The distillation time was 3 h at a rate of 3–4 mL/min. To improve consecutive chromatographic analyses, hexane was used instead of xylene. Each plant material was distilled five times to obtain a sufficient amount of essential oil for anti-cancer studies.

### 4.3. Making of Methanolic Dry Extracts

The methanolic extracts of the plant materials were prepared by double maceration by adding 200 mL of methanol to 10 g of crushed sample, which was allowed to stand in the dark at room temperature for 72 h. After filtration, the remaining plant material was returned to the flask and poured into 100 mL of methanol and allowed to stand in the dark for 24 h. After filtration, the two methanolic extracts were combined. The solvent was evaporated on a rotary evaporator in a water bath at 60 °C with a flask rotation of 70 rpm. The pressure was initially reduced to 300 mmHg, then the pressure was slowly increased to 10 mmHg until all the methanol had evaporated. The resulting dry extracts were stored in the freezer, and redissolved in 5 mL of methanol before analysis.

### 4.4. Gas-Chromatografic Analysis of the Essential Oils

The GC analysis of main compounds of essential oil was performed using Agilent (Santa Clara, CA, USA) GC 7890a chromatograph with software Agilent Open Lab CDS Chem Station and with FID on two fused silica capillary columns with stationary phases DB-5 and HP-Innowax (both 30 m × 0.25 mm, Agilent,). The carrier gas was hydrogen with split ratio 1:150 and the flow rate of 30 mL/min was applied. The temperature program was from 50 to 250 °C at 2.92 °C/min, and the injector temperature was 250 °C.

The identification of the oil components was accomplished by comparing their retention indices using Agilent Open Lab CDS Chem Station software. The content (%) of the components in the essential oils was determined from the mean retention times and peak areas of four parallel chromatograms. Components were identified by DB-5 column retention indices compared to databases and literature data.

### 4.5. High-Performance Liquid Chromatography of Polyphenolic Compounds

#### 4.5.1. Evaluation of Phenolic Profile of Plant Samples by MS Spectrum and MS/MS Fragment Analysis by LC–MS/MS Chromatography

Methanolic dry extract components were separated on Zorbax 300SB-C18 (2.1 × 150 mm; 5 µm, Agilent Technologies) reversed phase column (thermostated at +35 °C,). Mobile phase A: 0.1% formic acid in water; mobile phase B: acetonitrile. Flow rate 0.3 mL/min, sample size 5 µL and elution was performed according to stepwise gradient: 0–3 min B 1%; 3–40 min B 1–35%; 40–45 min B 35–95%; 45–50 min B 95%; 50.1 min B 1%.

Detection was performed by ion trap instrument (1100 series LC/MSD Trap-XCT, Agilent) with electrospray ionization in negative mode. Carrier gas was dry Helium (5.6 atm).

HPLC–MS/MS spectra were analyzed using HPLC-2D-ChemStation software. Polyphenolic components were identified by HPLC retention time and MS/MS fragments, comparing them with literature data and the inhouse database. More details are available in our previous paper (Rusalepp et al., 2017).

#### 4.5.2. Reagents

The reagents and reference substances were purchased from Sigma-Aldrich, Steinheim, Germany. Purified de-ionized water was prepared with the Milli–Q^®^ (Millipore, Bedford, MA, USA) water purification system.

#### 4.5.3. Evaluation of Phenolic Compound Profile by UPLC–MS/MS Conditions

Chromatographic separation of plant samples was performed on an Aquity H-class UPLC system (Waters, Milford, MA, USA) under conditions described by González-Burgos et al. [30]. A YMC Triart C18 (100 × 2.0 mm 1.9 µm) column was used for separation of phenolic compounds. The column temperature was maintained at 40 °C. Gradient elution was performed with mobile phase consisting of 0.1% formic acid water solution (solvent A) and acetonitrile (solvent B) with the flow rate set to 0.5 mL/min. A linear gradient profile was applied with following proportions of solvent A: 0 to 1 min—95%, 5 min.—70%, 7 min. 50%, 7.5 to 8 min. 0%, 8.1 to 10 min. 95%. MS and MS/MS analyses of separated peaks of phenolic compounds were carried out with triple quadrupole tandem mass spectrometer (Xevo, Waters, USA). An electrospray ionisation source (ESI) was used to obtain negative ions. Electrospray ionization was applied for analysis with the following settings: capillary voltage—2 kV, source temperature—150 °C, desolvation temperature—400 °C, desolvation gas flow—700 L/h, cone gas flow—20 L/h. Waters (USA) IntelliStart software was used for development of a specific collision energy and cone voltage for each compound of interest.

### 4.6. Cytotoxicity

Human cancer cell lines including hepatocellular carcinoma HepG2, gastric carcinoma MKN7, colon carcinoma SW480, prostate carcinoma LNCaP, and carcinoma in the mouth KB cells were cultured in DMEM or RPMI-1640 cell culture medium, both supplemented with 10% fetal bovine serum. Cells were cultivated at 37 °C in a humidified atmosphere containing 5% carbon dioxide.

The methanolic dry extracts were dissolved in dimethyl sulfoxide (DMSO) to prepare 4 mg/mL stock solutions that were later mixed with cell culture medium to achieve the desired concentrations. The final test concentrations were 0.8, 4, 20, and 100 μg/mL.

The effects of essential oils and extracts on viability of malignant cells were determined by sulforhodamine B cytotoxic assay [31]. Briefly, cells were grown in 96-well microtiter plates with each well containing 190 μL of medium. After 24 h, 10 μL of test samples dissolved in DMSO were added to each well. One plate with no samples served as a day 0 control. The cells were continuously cultured for an additional 48 h, fixed with trichloroacetic acid, and stained with sulforhodamine B, followed by the determination of optical densities at 515 nm using a Microplate Reader (BioRad, Hercules, CA, USA). The percentage of growth inhibition was calculated using the following equation:

$$\%\;\mathrm{Growth}=\frac{\left[\mathrm{OD}\;\left(\mathrm{reagent}\right)-\mathrm{OD}\;\left(\mathrm{day}\;0\right)\right]\times 100}{\left[\mathrm{OD}\;\left(\mathrm{negative}\;\mathrm{control}\;\mathrm{DMSO}\;10\%\right)-\mathrm{OD}\;\left(\mathrm{Day}\;0\right)\right]}$$

where OD is optical density or absorbance values. The potent anticancer agent ellipticine was used as a positive control.

## 5. Conclusions

Z- and E-ocimenes, isoborneol, crysanthenyl acetate, humulene epoxide, 2-pentadecanone, and nerolidon acetate were found in the essential oil of *A. tinctoria* for the first time.

The total polyphenol content of aerial parts of *A.tinctoria* was about 3.1 and 4.5 times higher than of aerial parts of *A. tinctoria* and roots of *A. sylvestris*, respectively. Total content of flavonol glycosides of aerial parts of *A. tinctoria* was about 4 and 8 times higher than in aerial parts of *A. tinctoria* and roots of *A. sylvestris.*

The principal polyphenols in the aerial parts of both *A. tinctoria* and *A. sylvestris* were caffeoylquinic and dicaffeoylquinic acids as well as several glycosides of quercetin and patuletin. In the aerial parts of *A. Sylvestris,* chlorogenic and caffeoylquinic acids and different glycosides of quercetin were also found.

The strongest anticancer effects were found in *A. sylvestris* roots’ and aerial parts’ essential oils on KB cells (IC_50_ 19.73 μg/mL and 19.84 μg/mL, respectively), and strong to moderate effects on other cell lines with IC_50_ ranges 24.69–38.06 μg/mL. The essential oil of *A. tinctoria* showed a strong effect on KB and LNCaP cells (IC_50_ between 27.75 μg/mL and 29.96 μg/mL, respectively). The methanolic extract of aerial parts of both plants had no effect on cancer cells (IC_50_ > 100 μg/mL). The essential oils of *A. tinctoria* and *A. sylvestris* may have some perspective in development of natural anticancer compounds.

## Figures and Tables

**Table 1:** Essential oil content of aerial parts of *Anthemis tinctoria*.

Compound	RI * (DB-5)	Content in Essential Oil (%)
Sabinene	972	0.7
β-Pinene	975	2.7
Myrcene	983	1.0
p-Cymene	1019	12.6
(Z)-β-Ocimene	1040	4.3
(E)-β-Ocimene	1048	2.6
Isoborneol	1147	2.1
Terpinen-4-ol	1174	4.0
Crysanthenyl acetate	1260	3.6
δ-Cadinene	1520	1.6
Caryophyllene oxide	1572	2.8
Isocaryophyllene oxide/caryophyllenol	1577	3.9
Humulene epoxide	1603	2.9
δ-Cadinol	1638	6.6
α-Muurolene	1648	12.5
2-Pentadecanone	1680	2.5
Nerolidol acetate	1720	2.5
n-Hexadecanal	1814	2.5
Palmitic acid	1967	15.3
Unknown	2534	12.6
Total		99.3
Monoterpenes		15.5
Cyclic monoterpenes		5.5
Bicyclic sesquiterpenes		6.7
Sesquiterpenes		24.5
Other compounds		47.1

* RI, retention index.

**Table 2:** Essential oils content of aerial parts and roots of *Angelica sylvestris*.

Compound	RI * (DB-5)	Content in Essential Oils (%)	
		Aerial Parts	Roots
α-Pinene	933	45.4	12.4
Camphene	946	4.6	nf
Sabinene	971	1.1	nf
β-Pinene	974	2.0	nf
β-Myrcene	990	13.3	nf
α-Terpinen	1014	0.4	0.7
p-Cymene	1018	15.5	8.2
β-Phellandrene	1030	1.3	nf
(Z)-β-Ocimene	1040	1.1	0.9
(E)-β-Ocimene	1048	0.7	nf
Terpinolene	1085	1.2	nf
n-Nonanal	1108	nf	1.3
α-Terpineol	1188	nf	1.2
(E)-Verbenyl acetate	1301	1.4	nf
β-Elemene	1391	nf	1.2
(E)-β-Caryophyllene	1416	0.8	nf
β-Copaene	1424	nf	2.7
α-Humulene	1450	1.0	nf
β-Farnesene	1456	nf	2.2
Germacrene D	1477	3.2	nf
β-Bisabolene	1501	nf	1.5
γ-Cadinene	1507	0.8	nf
Cadina-1,4-diene	1536	nf	2.3
Elemol	1546	nf	3.4
Caryophyllene oxide	1573	nf	2.4
Isocaryophyllene oxide/caryophyllenol	1582	nf	31.9
Epiglobulol/humulene epoxide	1614	nf	1.4
α-Muurolene	1648	nf	4.2
α-Cadinol	1663	nf	1.7
α-Bisabolol	1680	1.4	17.5
Nerolidol acetate	1721	nf	1.3
Unknown	1857	nf	1.5
Palmitic acid	1965	3.3	nf
Total		99.9	99.9
Monoterpenes		16.7	2.8
Cyclic monoterpenes		49.8	12.4
Bicyclic sesquiterpenes		0.8	34.3
Sesquiterpenes		6.4	36.7
Other compounds		24.8	13.7

* RI, retention index; nf, not found.

**Table 3:** Polyphenolic compounds identified in the methanolic extracts of *A. tinctoria* and *A. sylvestris* by HPLC-ion trap MS/MS.

t_R_ (min)	[M-H]^−^	MS/MS	Plant Material/Substance		
			Aerial Parts of *A. tinctoria*	Aerial Parts of *A. sylvestris*	Roots of *A. sylvestris*
0.5	341	179, 161	Caffeic acid glucosides	Caffeic acid glucosides	Caffeic acid glucosides
1.7	315	225, 153, 109	Protocatechuic or gentisic acid glucoside	Protocatechuic or gentisic acid glucoside	nf
4.3	325	163; 119	nf	nf	Coumaric acid glucoside
5.1	299	137	4-Hydroxybenzoic acid glucoside	4-Hydroxybenzoic acid glucoside	nf
10.3	339	281, 251, 177, 135	Daphnin = daphnetin glucoside	nf	nf
12.5	353	191, 179, 173, 135	Neochlorogenic acid	Neochlorogenic acid	Neochlorogenic acid
15.2	385	223, 179, 163	Sinapinic acid glucoside	nf	nf
15.5	353	306, 191, 135	nf	Chlorogenic acid	nf
16.5	639	463, 301, 535	Quercetin glucoside glucuronide	nf	nf
16.7	337	191, 163, 173	nf	Coumaroylquinic acid	Coumaroylquinic acid
17.3	625	463, 301	Quercetin diglucoside	nf	nf
17.8	335	179, 135	Caffeoylshikimic acid	nf	nf
18.6	367	191, 173	5-Feruloylquinic acid	5-Feruloylquinic acid	5-Feruloylquinic acid
19.8?	479	317	Myricetin glucoside	nf	nf
20.9	625	301	nf	Quercetinlucoside-glucoside	nf
21.3	655	493, 331	Patuletin diglucoside	nf	nf
22.0	449	287, 151	Eriodictyol glucoside	nf	nf
22.2	741	609, 475, 343, 301	Quercetin rutinoside pentoside	nf	nf
22.6	477	301, 373	Quercetin glucuronide	nf	nf
22.8	463	301, 179, 343	Quercetin galactoside	Quercetin galactoside	nf
23.2	609	301, 343, 271	Rutin	Rutin	Rutin, traces
23.3	463	301	Quercetin glucoside	Quercetin glucoside	Quercetin glucoside
24.0	493	331, 373	Patuletin glucoside	nf	nf
24.9	477	315, 433	Isorhamnetin glucoside	nf	nf
24.9	505	463, 301, 179, 151	Quercetin acetyl glucoside	nf	nf
25.4	515	353, 191	Dicaffeoylquinic acid 1	Dicaffeoylquinic acid 1	nf
26.4	493	331, 287	Patuletin-7-glucoside	nf	nf
26.4	373	211, 193	Pinosylvin glucoside	nf	nf
26.7	477	315, 357, 300	Isorhamnetin glucoside	Isorhamnetin glucoside	nf
27.3	607	299, 284	Diosmetin rutinoside = diosmin	Diosmetin rutinoside = diosmin	nf
27.4	515	191, 179, 255, 299, 353	3,4-Dicaffeoylquinic acid-2	3,4-Dicaffeoylquinic acid-2	3,4-Caffeoylquinic acid
27.7	489	285	Kaempferol acetylglucoside	nf	nf
29.1	529	353, 191	Feruloylquinic acid glucoside	Feryloylquinic acid glucoside	nf
30.4	535	331, 316	Patuletin acetylglucoside	nf	nf
31.0	331	316, 209	Patuletin	nf	nf
31.8	315	300	Iso-rhamnetin	nf	nf
32.6	271	151, 177, 119	nf	Naringenin	Naringenin
32.8	609	285	Kaempferol rutinoside	nf	nf
33.0	345	330, 315	Spinacetin	nf	nf
34.1	677	515, 353, 255, 191	Tricaffeoylquinic acid	Tricaffeoylquinic acid	nf
34.3	271	107, 119, 151	nf	nf	Naringenin chalcone
35.3	299	284	Diosmetin	nf	nf
35.7	315	300, 251	Nepetin	nf	nf
36.6	329	314, 171	Jaceosidin	nf	nf
38.1	359	344, 329	Jaceidin	nf	nf
38.3	593	447; 301	nf	Quercetin dirhamnoside	nf
38.4	593	285	nf	Luteolin rutinoside	nf

t_R_, retention time; [M-H]^−^, ion mass; MS/MS, mass of identified fragments; nf, not found.

**Table 4:** Polyphenolic compounds identified in the methanolic extract of *A. tinctoria* and *A. sylvestris* by the UPLC–triple quadrupole MS/MS (MRM) method.

t_R_ (min)	Precursor Ion (*m*/*z*) [M-H]^−^	Product Ion (*m*/*z*) MS/MS	Aerial Parts of *A. tinctoria*	Aerial Parts of *A. sylvestris*	Roots of *A. sylvestris*
0.46	191	85	Quinic acid	Quinic acid	Quinic acid
2.80	353	191	Neochlorogenic acid	Neochlorogenic acid	Neochlorogenic acid
3.75	353	191	Chlorogenic acid	Chlorogenic acid	Chlorogenic acid
3.98	179	107	Caffeic acid	Caffeic acid	nf
5.06	609	300	Rutin	Rutin	nf
5.15	593	285	Luteolin 7-rutinoside	Luteolin 7-rutinoside	Luteolin 7-rutinoside
5.18	193	134	Ferulic acid	Ferulic acid	Ferulic acid
5.39/5.55/5.76	515	353	Dicaffeoylquinic acids	Dicaffeoylquinic acids	Dicaffeoylquinic acids
5.22	463	301	Hyperoside	nf	nf
5.26	447	285	Luteolin-7-glucoside	nf	nf
5.28	463	301	Isoquercitrin	Isoquercitrin	Isoquercitrin
5.52	623	315	Isorhamnetin 3-rutinoside	nf	nf
5.70	447	300	Quercitrin	Quercitrin	Quercitrin
5.70	447	285	Luteolin 4-glucoside	nf	nf
6.79	285	133	Luteolin	nf	nf
6.86	301	151	nf	Quercetin	nf
7.22	271	151	nf	Naringenin	Naringenin
7.38	269	117	Apigenin	Apigenin	Apigenin
7.57	299	284	nf	Diosmetin	Diosmetin
7.60	315	300	nf	Isorhamnetin	nf

t_R_, retention time; [M-H]^−^, ion mass; MS/MS, mass of identified fragments; nf, not found.

**Table 5:** Total phenolics in mg/g of respective plant material, estimated by area under UV–chromatogram (AUC) at 280 nm and total chlorogenic acid and derivatives in mg/g, estimated by AUC at 330 nm, using chlorogenic acid as standard in both cases, and total flavonol content by area under the chromatogram at 360 nm which is specific for the flavonols absorption spectrum maximum.

Plant Part	Total Phenolics	Total Chlorogenic Acids	Total Flavonols, 360 nm
*A. tinctoria*, herb	14.7	12.7	11,471
*A. sylvestris,* herb	5.5	4.9	2629
*A. sylvestris*, root	2.3	2.1	0

**Table 6:** Total quinic acid derivatives, estimated as total area of MS2 = 191 peaks on LC–MS chromatograms in MS count units.

Plant Part	Total Quinic Acid Derivatives
*A. tinctoria*, herb	320,218
*A. sylvestris,* herb	259,839
*A. sylvestris*, root	105,600

**Table 7:** Anticancer activity of essential oils and methanolic extracts of *A. tinctoria* and *A. sylvestris*.

Concentration (µg/mL)	% Inhibition				
	Essential oil of *A. tinctoria* (Aerial Parts)				
	HepG2	MKN7	SW480	LNCaP	KB
100	91.80	97.07	81.11	93.85	96.32
20	25.33	28.47	23.85	38.51	40.18
4	12.88	19.99	17.81	28.31	25.99
0.8	0.13	8.81	8.59	12.23	10.03
IC_50_	44.98 ± 2.96	43.04 ± 4.50	55.45 ± 5.70	29.96 ± 2.25	27.75 ± 1.86
Concentration (µg/mL)	Essential oil of *A. sylvestris* (aerial parts)				
	HepG2	MKN7	SW480	LNCaP	KB
100	87.08	88.79	103.03	90.21	96.76
20	33.14	32.31	38.47	42.23	52.30
4	11.61	15.34	13.35	20.35	18.36
0.8	−2.02	1.93	4.45	7.21	4.92
IC_50_	37.46 ± 2.33	38.06 ± 2.09	30.72 ± 1.81	27.78 ± 1.28	19.84 ± 2.35
Concentration (µg/mL)	Essential oil of *A. sylvestris* (roots)				
	HepG2	MKN7	SW480	LNCaP	KB
100	97.82	99.34	75.35	95.08	97.22
20	44.11	36.14	40.70	39.34	49.19
4	20.33	12.44	27.66	21.86	28.60
0.8	5.14	6.00	16.85	10.53	14.28
IC_50_	24.69 ± 1.96	34.09 ± 2.08	33.36 ± 2.25	30.37 ± 2.35	19.73 ± 2.18
Concentration (µg/mL)	Methanolic extract of *A. tinctoria* (aerial parts)				
	HepG2	MKN7	SW480	LNCaP	KB
100	21.29	30.50	29.89	29.83	25.36
20	14.28	8.76	5.09	8.83	10.46
IC_50_	>100	>100	>100	>100	>100
Concentration (µg/mL)	Methanolic extract of *A. sylvestris* (aerial parts)				
	HepG2	MKN7	SW480	LNCaP	KB
100	14.50	29.69	20.61	36.32	19.62
20	2.91	9.65	7.00	7.85	8.12
IC_50_	>100	>100	>100	>100	>100
Concentration (µg/mL)	Methanolic extract of *A. sylvestris* (roots)				
	HepG2	MKN7	SW480	LNCaP	KB
100	67.48	68.94	63.43	76.48	73.07
20	26.01	25.71	22.73	34.81	36.29
4	12.11	7.09	11.76	17.33	19.60
0.8	−0.60	0.66	−1.11	4.57	8.14
IC_50_	57.37 ± 3.57	58.52 ± 3.52	66.06 ± 2.74	40.08 ± 2.22	40.60 ± 1.85
Concentration (µg/mL)	Ellipticine *				
	HepG2	MKN7	SW480	LNCaP	KB
10	103.80	97.28	87.44	93.85	99.82
2	86.90	88.59	78.68	80.01	78.04
0.4	49.17	47.09	48.76	49.33	51.45
0.08	22.02	20.66	21.11	24.57	28.34
IC_50_	0.38 ± 0.04	0.41 ± 0.05	0.48 ± 0.05	0.40 ± 0.04	0.35 ± 0.02

HepG2, human hepatocellular carcinoma; MKN7, human gastric carcinoma; SW480, human colon carcinoma; LNCaP, human prostate carcinoma; KB, human carcinoma in the mouth. Ellipticine: the positive control sample; * the positive control sample.

## Data Availability

The data presented in this study are available in article and Supplementary Materials.

## Supplementary Materials

The following supporting information can be downloaded at: https://www.mdpi.com/article/10.3390/plants11070994/s1, Figure S1: HPLC-chromatograms of aerial parts of Anthemis tinctoria, Figure S2: HPLC-chromatograms of aerial parts of Angelica sylvestris, Figure S3: HPLC-chromatograms of roots of Angelica sylvestris.
