# Supplementary figures and images for: The Phytochemical Profile and Anticancer Activity of Anthemis tinctoria and Angelica sylvestris Used in Estonian Ethnomedicine
Source: Plants (Basel). 2022 Apr 5;11(7):994. doi: 10.3390/plants11070994 (PMC9003001; doi:10.3390/plants11070994)

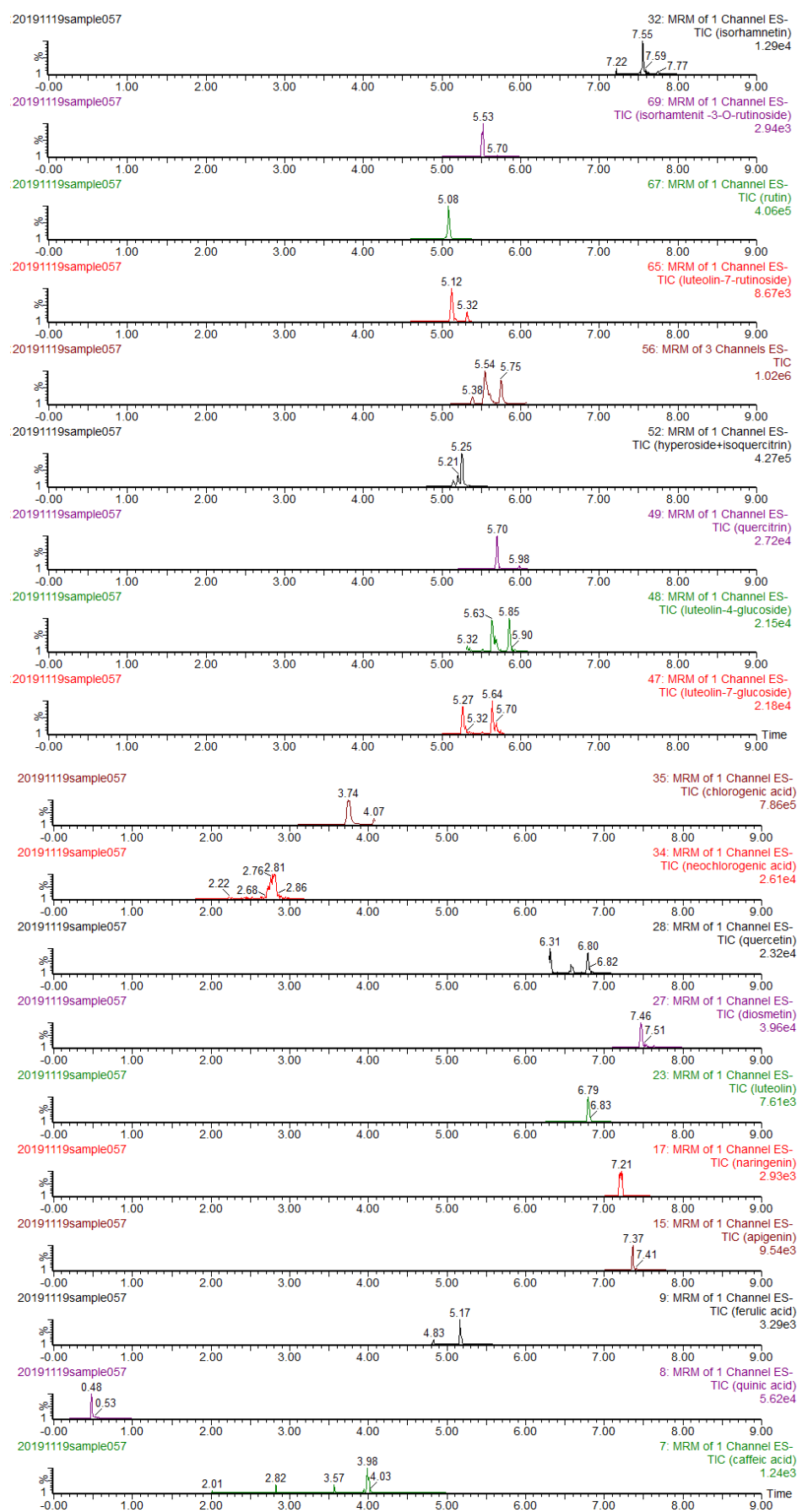

**Figure S1.** HPLC-chromatograms of aerial parts of *Anthemis tinctoria*.

Supplement: Supplementary file 1 [file plants-11-00994-s001.zip › plants-1670305-supplementary/Figure S1.pdf]

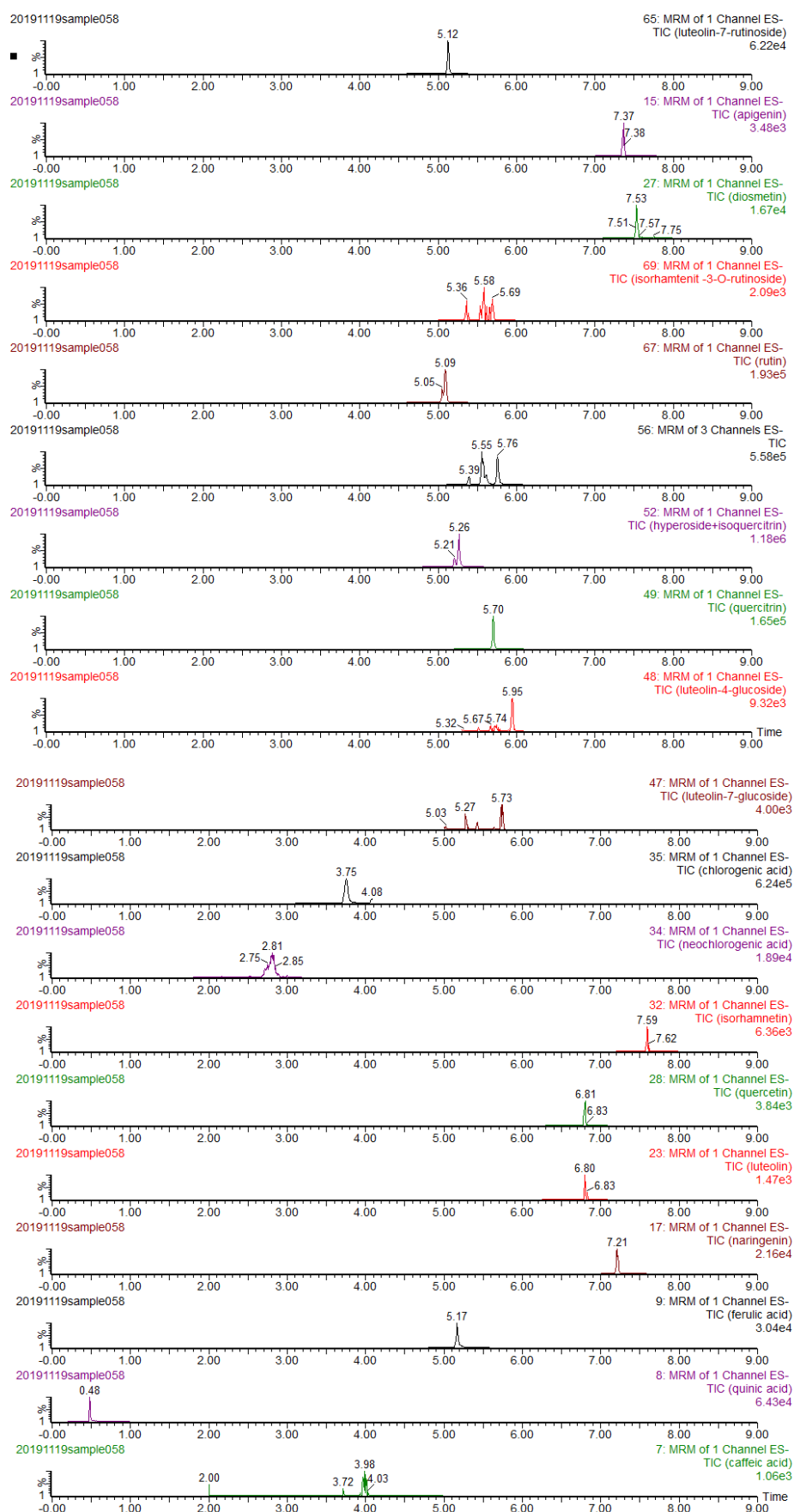

**Figure S2.** HPLC-chromatograms of aerial parts of *Angelica sylvestris*.

Supplement: Supplementary file 1 [file plants-11-00994-s001.zip › plants-1670305-supplementary/Figure S2.pdf]

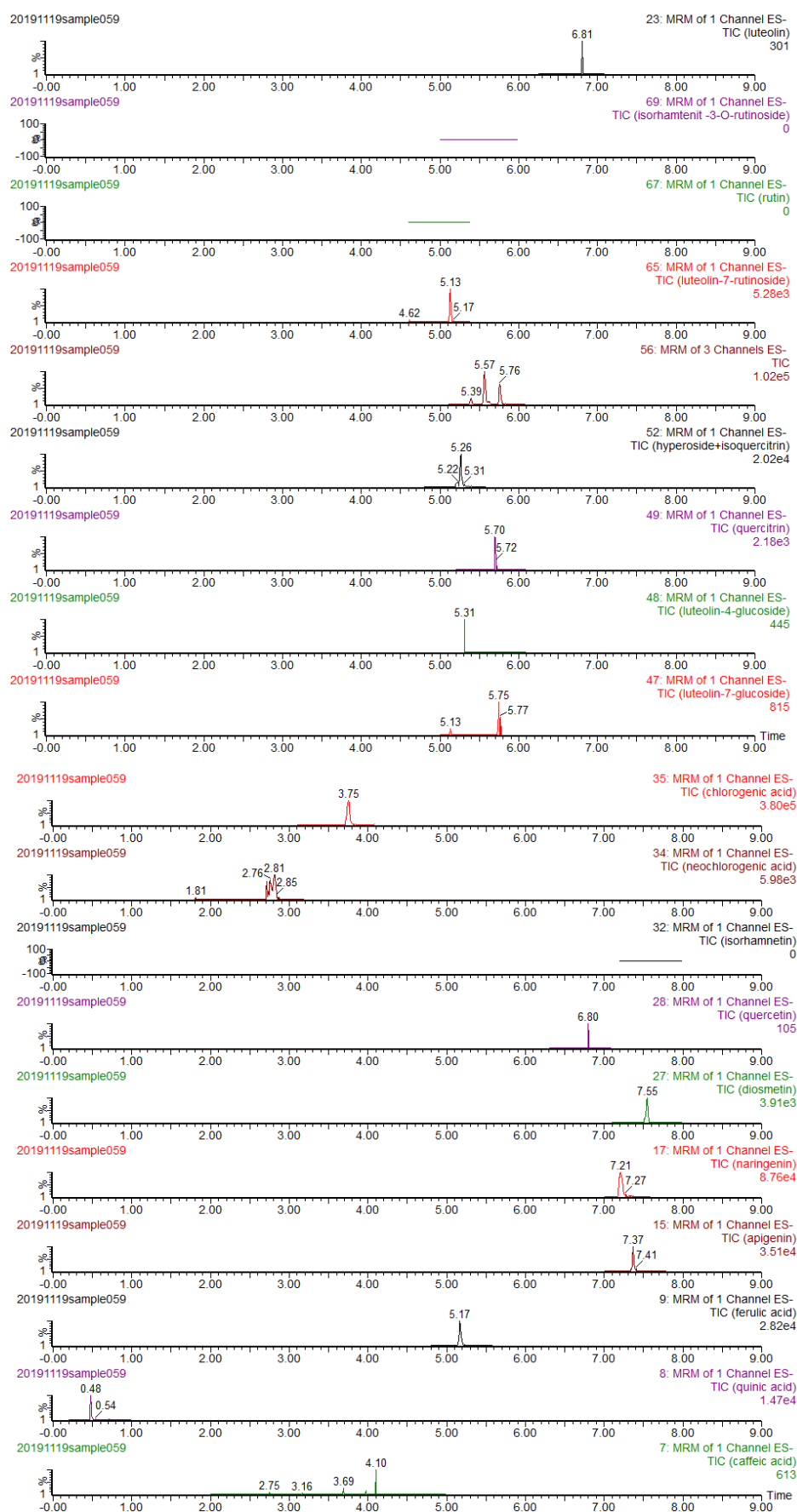

**Figure S3.** HPLC-chromatograms of roots of *Angelica sylvestris*.

Supplement: Supplementary file 1 [file plants-11-00994-s001.zip › plants-1670305-supplementary/Figure S3.pdf]
